# Impact of Probiotics on the Prevention and Treatment of Gastrointestinal Diseases in the Pediatric Population

**DOI:** 10.3390/ijms24119427

**Published:** 2023-05-29

**Authors:** José Antonio García-Santos, Ana Nieto-Ruiz, María García-Ricobaraza, Tomás Cerdó, Cristina Campoy

**Affiliations:** 1 Department of Paediatrics, School of Medicine, University of Granada, Avda. Investigación 11, 18016 Granada, Spain; joseantonio_gsantos@outlook.es (J.A.G.-S.); ananietoruiz@gmail.com (A.N.-R.); mariaricobaraza@ugr.es (M.G.-R.); 2 EURISTIKOS Excellence Centre for Paediatric Research, Biomedical Research Centre, University of Granada, Avda del Conocimiento 19, 18016 Granada, Spain; 3 Instituto de Investigación Biosanitaria de Granada (ibs-GRANADA), Health Sciences Technological Park, Avda. de Madrid 15, 18012 Granada, Spain; 4 Maimonides Institute for Research in Biomedicine of Córdoba (IMIBIC), Av. Menéndez Pidal, s/n, 14004 Córdoba, Spain; 5 Centre for Rheumatology Research, Division of Medicine, University College London, Gower Street, London WC1E 6BT, UK; 6 Spanish Network of Biomedical Research in Epidemiology and Public Health (CIBERESP), Granada’s Node, Carlos III Health Institute, Avda. Monforte de Lemos 5, 28028 Madrid, Spain

**Keywords:** functional gastrointestinal disorders, gastrointestinal disorders, gut microbiota, probiotics, infants, children

## Abstract

Despite the high prevalence of gastrointestinal disorders (GIDs) in infants and children, especially those categorized as functional GIDs (FGIDs), insufficient knowledge about their pathophysiology has limited both symptomatic diagnosis and the development of optimal therapies. Recent advances in the field of probiotics have made their potential use as an interesting therapeutic and preventive strategy against these disorders possible, but further efforts are still needed. In fact, there is great controversy surrounding this topic, generated by the high variety of potential probiotics strains with plausible therapeutic utility, the lack of consensus in their use as well as the few comparative studies available on probiotics that record their efficacy. Taking into account these limitations, and in the absence of clear guidelines about the dose and timeframe for successful probiotic therapy, our review aimed to evaluate current studies on potential use of probiotics for the prevention and treatment of the most common FGIDs and GIDs in the pediatric population. Furthermore, matters referring to know major action pathways and key safety recommendations for probiotic administration proposed by major pediatric health agencies shall also be discussed.

## 1. Introduction

Clinical approaches have traditionally focused attention on pediatric functional gastrointestinal diseases (FGIDs), which are characterized by recurring gastrointestinal symptoms, including abdominal pain, vomiting or constipation, that can ultimately cause non-optimal development, disrupt digestion or create lifelong or mortal complications. In addition, there is a growing body of evidence suggesting significant prevalence and persistent comorbidities in pediatric patients with organic gastrointestinal diseases (GIDs) [1,2]. Notwithstanding the abovementioned considerations, major efforts are still needed to better understand complex pathophysiological mechanisms involved in GIDs, which will undoubtedly lead to the development of new therapeutic approaches [3]. At this time, scientific evidence suggests that both GIDs and FGIDs are related to gut microbial dysbiosis, gastrointestinal motility disturbance, visceral hypersensitivity, as well as impairment of mucosal immune function and central nervous system processing [1]. Based on this, the use of probiotic bacteria, mainly those strains belonging to *Lactobacillus*, *Bifidobacterium* and probiotic yeast such as *Saccharomyces*, have emerged as potential therapeutic agents in GIDs [4,5,6]. These clinical benefits are largely based on their multiple mechanisms of action, including immunomodulatory and anti-inflammatory properties, improvement of intestinal mucosal barrier function and inhibition of potential pathogen adhesion [7]. However, there is limited information about the effective doses and treatment duration, and current recommendations only provide levels of evidence for probiotic treatment benefits in accordance with the Oxford Centre for Evidence-Based Medicine criteria (Table 1) [8], as well as the evidence-based pediatric guideline for pro, pre and synbiotics in gastroenterology provided by the World Gastroenterology Organisation (Table 2) [8].

Far from being a clinical guideline, this review aimed to explore and summarize the current knowledge about probiotics’ mechanisms of action, effective doses and safety conditions in their use for the prevention and treatment of the most common organic (acute infectious diarrhea, antibiotic-associated diarrhea, helicobacter pylori infection, necrotizing enterocolitis, inflammatory bowel disease and prevention of food allergy) and functional GIDs (infant colic, functional constipation, irritable bowel disease, functional regurgitation, cycling vomiting and functional dyspepsia) in infants and children. Nevertheless, although further experimental and clinical studies are still needed, better knowledge surrounding this promising scientific area will be useful to reduce both the incidence and severity of pediatric GIDs, thus improving quality of life, short- and long-term, of infants and children. In order to achieve these purposes, a comprehensive search of published original scientific studies, high evidence systematic reviews and meta-analysis and practice guidelines dating from 2018 to 2023 was carried out using Google Scholar, PubMed and Cochrane Library databases. Moreover, relevant scientific papers before the abovementioned dates were also included. The main search terms used were [“probiotic” OR “prebiotic” OR “symbiotic” OR “postbiotic” OR “parabiotic”] AND [“infants” OR “children” OR “paediatric population” OR “preterm infants] for each specific gastrointestinal or functional gastrointestinal disorder mentioned above. Studies carried out in animal models were used to better explain the potential mechanisms of action of pro-, pre-, post- or parabiotics in functional or gastrointestinal disorders. These studies were also included to suggest the potential beneficial effects of these treatments in cases where evidence in human patients is limited. Those studies unrelated to the rationale of the current review or having low-scientific evidence, both in terms of animal models and human patients, were excluded.

## 2. Mechanisms of Action of Probiotics

**Figure 1:**
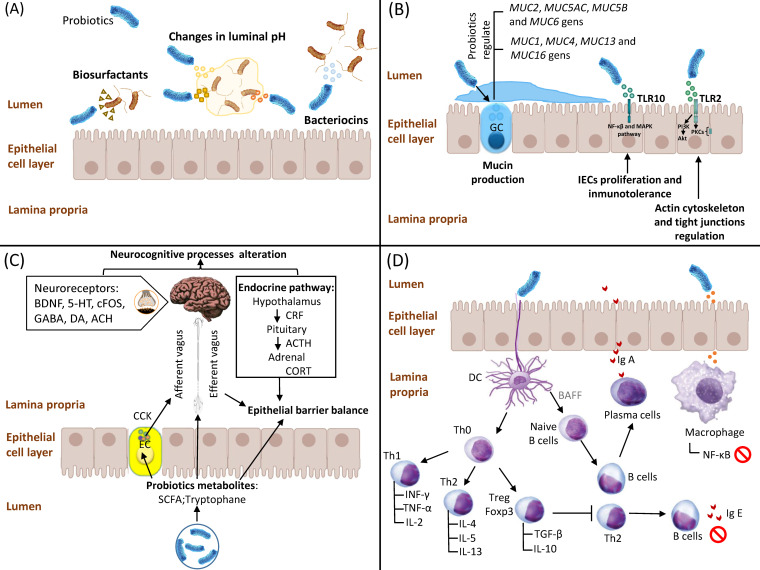
Mechanisms of action of probiotics. (**A**) Competitive adhesion and exclusion of potential pathogens. Probiotics create a hostile environment via changes in luminal pH as well as production of bacteriocins and biosurfactants. (**B**) Stimulation of intestinal epithelial cell (IEC) proliferation and epithelial barrier enhancement. Probiotics modulate IECs-dependent mucin production through regulation of several *MUC* genes. Moreover, probiotics enhance epithelial barrier and tight junctions due to their modulation of the NF-κB, MAPK PI3K/Akt and PKCs signalling pathways. (**C**) Interaction of probiotics and their metabolites (mainly short-chain fatty acids (SCFAs) and tryptophane) with the enteric nervous system. Probiotics modulate neurocognitive processes and epithelial barrier balance via vagus and enteric nerves; probiotics also affect the hypothalamic–pituitary–adrenal (HPA) axis via the regulation of several neurotransmitter levels, including CORT and/or ACTH, BDNF, 5-HT, c-Fos, DA, ACh and GABA. (**D**) Interaction of probiotics with the immune system: (i) regulation of Th1/Th2 cytokine balance and activation of CD4+ Foxp3+ Treg cells; (ii) increased levels of B cell-dependent secretory IgA (sIgA) and concomitant reduction in IgE levels; and (iii) downregulation of NF-κB signalling pathway and subsequent inhibition of inflammatory cytokines expression. Abbreviations: GC: goblet cell; PI3K: phosphatidylinositol 3-kinase; PKC: protein kinase C; EC: enteroendocrine cells; SCFA: short-chain fatty acid; CCK: cholecystokinin; CRF: corticotropin-releasing factor; ACTH: adrenocorticotropic hormone; CORT: corticosteroid; BDNF: brain-derived neurotrophic factor; 5-HT: 5 hydroxytryptamine; GABA: γ-aminobutyric acid; DA: dopamine; ACH: acetylcholine; TLR: Toll-like receptor; TNF-α: Tumor Necrosis Factor α; IFN-γ: Interferon γ; TGF-β: Transforming Growth Factor β; IL-2: Interleukin 2; IL-4: Interleukin 4; IL-5: Interleukin 5; IL-10: Interleukin 10; IL-13: Interleukin 13; Th0: T Cell Naive; Th1: Type 1 Helper T cell; Th2: Type 2 Helper T cell; IgA: immunoglobulin A; IgE: immunoglobulin E; DC: dendritic cells; BAFF: B cell-activating factor.

As mentioned above, and irrespective of their classification as organic or functional, healthy maintenance of gut microbiota diversity can play a pivotal role in GIDs pathophysiology. Consequently, probiotics-based therapy has recently emerged as a promising strategy for the prevention and treatment of these diseases [9]. Although the mechanisms of action of probiotics are complex and may differ by species, there is documented evidence on various main action pathways, including competitive adhesion and exclusion of potential pathogens, stimulation of intestinal epithelial cell (IEC) proliferation and epithelial barrier enhancement, as well as their potential interaction with the enteric nervous system and immune system (Figure 1).

### 2.1. Competitive Adhesion and Exclusion of Potential Pathogens

Competitive exclusion is defined as a process by which one species of bacteria competes for the same receptor sites in the gastrointestinal tract (GIT) with other species, with identical needs for resources [10]. This mechanism could enable probiotic bacteria to prevent the proliferation of potential pathogens and their adhesion to the gut epithelia. In fact, probiotics have the ability to create a hostile GIT environment to pathogenic bacteria growth through different pathways, including: (1) changes in luminal pH mediated by the secretion of short-chain fatty acids (SCFAs), branched-chain fatty acids, hydrogen sulphide and organic acids such as lactate, succinate and phenylacetate [11]; (2) production of bacteriocins, mainly non-ribosomally synthesized antimicrobial peptides (e.g., gramicidin, bacitracin, polymyxin B and vancomycin) [12]; and (3) production of biosurfactants (rhamnolipids and surfactin) that drastically reduce the surface tension and interfacial tension at the air–water interfaces [13]. Finally, it is important to note that most bacteriocins and biosurfactants obtained from probiotic bacteria are related to a high number of lactic acid bacteria (LAB) [14,15].

### 2.2. Stimulation of IEC Proliferation and Epithelial Barrier Enhancement

IECs are non-hematopoietic cells with pleiotropic functions in relation to luminal microbiota regulation and the host immune system, acting as a physical barrier against external antigens [16]. Thus, IECs secrete a mucus coat mainly formed by glycocalyx covering the epithelial lining, which in turn creates a sheltering environment for commensal bacteria and provides an interface for immune response [17]. Scientific evidence suggests that probiotics may control IEC-mediated functions through different mechanisms, including: (1) modulation of mucin production via changes in the *MUC* gene expression profile [18]; (2) regulation of mucosal innate immunity and subsequent production of antiapoptotic and antioxidant proteins through Toll-like receptors (TLRs)/NF-κB and MAPK signalling pathways [19]; and (3) activation of TLR2s expressed in IECs, which leads to overexpression of phosphatidylinositol 3-kinase (PI3K)/Akt and protein kinase C (PKC) pathways, thus regulating the actin cytoskeleton and tight junctions in order to protect GI mucosal integrity [20].

### 2.3. Interaction of Probiotics with the Enteric Nervous System

In recent years, growing evidence obtained from animal studies suggests the potential modulatory role of gut microbiota on the gut–brain axis and brain function, thus supporting the use of probiotics in the treatment and prevention of several brain and gastrointestinal diseases [21]. Thus, SCFAs produced by commensal and probiotic bacteria play a pivotal role in gut microbiota–brain interactions due to their influence on the sympathetic nervous system [22], mucosal serotonin release [23] as well as memory and learning processes [24]. Moreover, other probiotic metabolites, such as tryptophan, butyrate and oleate, can also exert their functions on the enteric nervous system through vagus and enteric nerves [25]. In addition, probiotics can affect the hypothalamic–pituitary–adrenal (HPA) axis, inducing significant changes in corticosteroid (CORT) and/or adrenocorticotropic hormone (ACTH) levels. Finally, potential effects of probiotics on mind and behavior are also suggested due to the fact that their metabolism-derived compounds can modify neurotransmitters levels, including brain-derived neurotrophic factor (BDNF), 5 hydroxytryptamine (5-HT), c-Fos, dopamine (DA), acetylcholine (ACh) and γ-aminobutyric acid (GABA) [26]. However, it should be noted that those neurotransmitters mentioned are also synthesized by the gut microbiota, although they are functionally different from brain-derived neurotransmitters [27].

### 2.4. Interaction of Probiotics with the Immune System

Studies performed using next-generation analysis have also showed that probiotic-derived compounds regulate gene expression in immune cells and intestinal epithelium. This markedly increases immunoglobulin A (IgA) secretion by macrophages and/or dendritic cells, reduces lymphocyte polarization and cytokine profiles, as well as induces tolerance to food antigens [28]. In this sense, the immunomodulatory role of probiotics is mediated by different molecular pathways, including: (i) restoration of Th1/Th2 cytokine balance through the upregulation of Th2 cytokines (IL-4, IL-5, IL-13) [29], TGF-β and IL-10 production mediated by CD4+ Foxp3+ Treg cells [30]; (ii) increase in B cell-dependent secretory IgA (sIgA) [31] and concomitant reduction in allergen-specific IgE levels [32]; and (iii) downregulation of the NF-κB signalling pathway and subsequent inhibition of inflammatory cytokines expression [33]. However, these mentioned mechanisms are still not entirely clear, and further in vivo studies are needed to better understand interactions between probiotics as well as their derived compound and the immune system, which will allow us to develop novel therapeutic applications.

## 3. Safety of Probiotics

Traditionally, probiotics are defined as “non-pathogenic microorganisms strains mostly of human origin which, when administered in adequate amounts, have a proven beneficial effects on human health” [34]. However, these benefits largely depend on several factors, mainly the ability to survive the acidic conditions of the stomach, their viability in drug delivery vehicles and their capacity to adhere to the epithelial tissue [35]. Therefore, before they can be applied clinically, probiotic strains must overcome specific safety issues related to contamination or transferable antibiotic resistance genes; their effective doses, frequency, route and duration of administration; or intrinsic compositional characteristics to avoid potential allergic responses [36].

In 2011, the Agency for Healthcare Research and Quality (US) published a report based on a systematic review of 622 randomized controlled trials (RCTs) [37]. Despite evidence about their safety being limited, authors concluded that the majority of probiotic strains used were safe, and adverse effects were only reported in those patients with compromised health. Moreover, the incidence of adverse events was similar, regardless of the use of a single strain or probiotic strain mixture, but their long-term effects on human health were unclear. Recently, the European Paediatric Association/Union of National European Paediatric Societies (EPA/UNEPSA) convened a European expert panel to evaluate and review guidelines, position papers and offer current recommendations about the therapeutic use of probiotics in pediatric health care [38]. This document supports a strain-specific effective approach to preventing or treating certain diseases, including organic and functional GIDs. However, special caution should be taken regarding the use of probiotics in premature infants, immunocompromised and critically ill patients as well as those who require central venous catheters or suffer from cardiac valvular disease and short-gut syndrome.

To date, debate continues concerning probiotic-based therapy in preterm infants, especially considering the immaturity of their gastrointestinal tract and immune system. For that reason, Underwood et al. [39] summarized the main questions related to probiotic administration in preterm infants addressed at the Necrotizing Enterocolitis (NEC) Society Symposium in 2019, including a recent strain-specific network analysis as well as a position statement from the European Society for Paediatric Gastroenterology Hepatology and Nutrition (ESPGHAN). In summary, these authors concluded that probiotic administration has both potential benefits and risks for preterm infant health, the latter related to sepsis caused by the probiotic strain used or infections by probiotic product contamination. Taking into account these considerations, it is recommended that parents of premature infants are involved in the decision whether or not to follow a probiotic-based treatment, although there is consistent evidence that risks associated with probiotic treatment are low compared to its benefits on premature infant health.

## 4. Potential Role of Probiotics in the Prevention and Treatment of Prevalent Pediatric FGIDs

Throughout this section, we will proceed to summarize the current knowledge about the potential use of probiotics in the prevention and treatment of the most prevalent FGIDs in infants and children, showing a particular focus on effective doses and the probiotic strains used to achieve clinical improvement and symptom resolution.

### 4.1. Infant Colic

Infant colic is defined as “recurrent and prolonged periods of crying, fussing or irritability without evidence cause or other clinical signs reported by caregivers in infants under 5 months at the onset and resolution of symptoms” [40]. Although it has higher global prevalence during the first months of life, the underlying pathophysiology of infant colic has not been clearly established, and several gastrointestinal (immature gastrointestinal function, excessive intestinal gas, altered gut hormones levels, pathologic gastroesophageal reflux, or cow’s milk protein intolerance/allergy, among others) and non-gastrointestinal factors (mainly parental psychological issues) seem to be involved [41,42]. Interestingly, gut microbiota dysbiosis and related inflammatory status have emerged as novel pathophysiological mechanisms in infant colic. In this regard, infants suffering from colic show lower diversity and a different gut microbiota profile compared to those who are healthy. More specifically, colic-related dysbiosis is characterized by decreased relative abundance of *Bifidobacterium* spp. and *Lactobacillum* spp., as well as high levels of potentially pathogenic bacteria, including *Clostridioides*, *Staphylococcus* and *Enterobacteria* (mainly *Escherichia*, *Shigella*, *Klebsiella* and *Enterobacter*) [42,43]. It is also important to note that intestinal dysbiosis might exacerbate other potential causes of infant colic. For instance, altered gut microbiota could led to inappropriate fermentation of dietary lactose, carbohydrates and proteins, thus increasing the intra-gastrointestinal air load [44]. Moreover, through the gut microbiota–brain axis, this dysbiotic state also negatively influences central and enteric neuronal functions, increasing pain sensation and subsequent excessive crying [45]. However, further studies are needed to better understand whether gut dysbiosis causes or results in intestinal inflammation.

Due to its multifactorial aetiology, there is not a unique and effective therapy against infant colic. Classical therapeutic strategies are largely based on nutritional interventions using hydrolyzed formulas in formula-fed infants or low-allergen maternal diets in those who are breastfed [46]. On the other hand, non-nutritional therapies such as behavioral interventions, manual therapies and pharmacological treatments, show inconsistent results regarding their effectiveness as well as side effects, while the use of proton pump inhibitors is strongly discouraged [47]. Given the limited treatment options available, and in view of the pathophysiological role of gut microbiota dysbiosis in infant colic, probiotic therapy may be effective in improving relevant clinical outcomes. In fact, selected studies support probiotic treatment as the most effective therapy for reducing crying time in breastfed infants suffering from colic, which may be explained by its anti-inflammatory effects and protective role against gut dysbiosis [47]. To date, *Lactobacillus reuteri* (now *Limosilactibacillus reuteri*) is the major probiotic strain used for infant colic treatment, and its therapeutic use (alone or in combination with other probiotic strains such as *Lactobacilli* spp., *Bifidobacteria* spp. and *Streptococcus thermophilus*) significantly reduces colic symptoms after an intervention period between 5 to 28 days [48]. Once adhered to enterocytes via biofilm-forming exopolysaccharides production, *L. reuteri* exerts its probiotic effects through different mechanisms of action, including: (i) inhibition of pathogenic Gram-negative bacteria growth, mainly *Helicobacter pylori*, *Escherichia coli*, *Clostridioides difficile* and *Salmonella*, by producing several antimicrobial compounds and metabolites (reuterine, reuterocyclin, lactic acid, acetic acid, or ethanol, among others); (ii) modulation of the T cell-dependent immune response via increasing Foxp3 mRNA levels and Foxp3+ regulatory T cell populations in the ileum, which in turn reduces crying time in colicky infants; (iii) an anti-inflammatory effect related to the downregulation of pro-inflammatory cytokine expression (TNF-α and IFN-γ) and overproduction of anti-inflammatory mediators (IL-10, vitamins B9 and B12, as well as homeostatic CC-chemokine receptor 7); and finally, (iv) direct action on enteric nerves to decrease colic infant-associated visceral pain [48,49]. Recent approaches have also focused on evaluating the potential beneficial effects of other probiotic strains in infant colic treatment. For example, *Bifidobacterium breve* CECT7263-based therapy during 28 days significantly reduced crying time and fussing in colicky infants compared to the use of simethicone or a combined therapy based on *L. fermentum* CECT5716 and *B. breve* CECT7263 [50]. Similarly, administration of *Bifidobacterium animalis* subsp. *Lactis* BB-12 in colicky breastfed infants markedly decreased the number of crying episodes, thus improving both daily sleep duration and parental wellbeing [51]. Moreover, fecal samples from treated infants showed high levels of immunity biomarkers including β-defensin 2, cathelicidin, sIgA, calprotectin and butyrate, suggesting the potential immunomodulatory role of *Bifidobacterium animalis* subsp. *Lactis* BB-12 in the gut of infants suffering from colic. Taking all these considerations together, the current paper by the ESPGHAN Special Interest Group on Gut Microbiota and Modifications weakly recommends the use of *L. reuteri* DSM 17938 (10^8^ cfu/day for at least 21 days) or *B. lactis* BB-12 (10^8^ cfu/day, for 21–28 days) for the management of infant colic in breastfed infants, in combination with advice and assistance to parents [52]. However, no recommendations are established for their use in formula-fed infants, thus supporting the need to perform more well-designed studies, particularly among this population.

### 4.2. Functional Constipation

Functional constipation (FC) is a common pediatric health problem affecting about 3% of infants worldwide during their first year of life [53]. Its symptomatology is mainly characterized by infrequent and/or painful defecation, fecal incontinence episodes, abdominal pain and bloating, which negatively affect the infant’s quality of life [54]. Due to these symptoms possibly appearing without a clear organic cause, behavioral factors have been traditionally proposed as main triggers of FC [55,56]. Unfortunately, some of the symptoms remain during early childhood and adulthood, suggesting a multifactorial pathophysiology of FC involving unknown genetic factors, unhealthy lifestyles related to poor dietary and exercise habits, as well as abnormal physiological characteristics (reduced number of interstitial cells of Cajal and low substance P and vasoactive intestinal peptide levels) [57,58].

Current scientific interest has focused on the role of gut microbiota dysbiosis and altered gastrointestinal motility in the emergence and development of this disorder. Despite a common microbial profile not being observed in FC patients, the data achieved suggest a low abundance of *Bifidobacteria* (*B. longum*), *Lactobacillus*, *Bacteroides* (*B. fragilis* and *B. ovatus*), *Prevotella* and *Alistipes finegoldii*, while abundances of *Parabacteroides* spp., *Clostridia* spp. as well as bacteria belonging to *Coprococcus*, *Ruminococcus*, *Blautia*, and *Anaerotruncus* genera seem to be increased in constipated patients [59,60]. These data also suggest a variety of potential molecular and biochemical mechanisms through which gut microbiota dysbiosis causes functional constipation. First, gut microbiota composition may alter bile acid (BA) profile in infants with constipation by regulating cholesterol 7α-hydroxylase (CYP7A1)-dependent de novo BAs synthesis and/or increasing its sulfation [61,62]; ultimately, this altered BA profile negatively affects both intestinal motility and colonic fluid. Another of the mechanisms proposed is related to SCFA production, mainly acetate, propionate and butyrate, via the bacterial fermentation of non-digestible carbohydrates. In this regard, studies conducted support evidence of a high abundance of butyrate-producing bacteria communities in the gut microbiota of FC patients, which might favor its pathogenesis by increasing electrolyte absorption and reducing mucin secretion [60,63]. Moreover, FC-related dysbiosis can lead to an imbalance between SCFA production and colonic absorption, affecting gut motility and intestinal transit. This imbalance is mediated by different mechanisms of action, including: (i) an increase in intestinal pH; (ii) a regulatory effect on the production of serotonin (5-HT), peptide YY and glucagon-like peptide-1 in enterochromaffin cells; and (iii) the potential activation of intrinsic primary afferent neurons via free fatty acid (FFA) receptors FFA1 and FFA2 [64]. As noted above, regulation of 5-HT secretion by gut microbiota has been also proposed as an additional mechanism to control gut motility. This effect seems to be mainly mediated through gene expression modulation of a serotonin transporter whose upregulation in FC patients causes gut 5-HT depletion and slow colonic transit [65]. Interestingly, other studies also suggest that 5-HT depletion could be related to altered tryptophan metabolism in terms of overproduction of indole and kynurenine [66]. Finally, gut microbiota from FC patients is enriched with methanogenic bacteria, and resultant excessive methane production leads to increasing colonic transit time and reducing the number of bowel movements [67].

Regarding the management and treatment of FC in infants and children, North American Society for Pediatric Gastroenterology, Hepatology and Nutrition (NASPGHAN) and ESPGHAN guidelines recommend the importance of adopting healthy life habits and pharmacologic treatment for rectal fecal disimpaction accompanied by specific behavioral protocols [58]. Despite these recommendations, the health care burden of FC is substantial, which supports the need to explore and evaluate novel therapeutic strategies. Among them, probiotic treatment has emerged as a promising tool in the prevention and treatment of pediatric FC. In fact, probiotic administration may exert a modulatory effect on the colonic mucosal microbiota composition related to FC; however, results achieved to date suggest the lack of compositional changes or a probiotic strain-specific impact on certain microbiota components, mainly *Bifidobacteria* [68,69,70]. Similar inconsistent results have been obtained in evaluating the effects of probiotics on metabolic microbial via products such as SCFAs [71]. In addition to their abovementioned potential beneficial effects on FC pathogenesis, SCFAs also seem to show intestinal regulatory T cell-mediated anti-inflammatory activity through a suppression of hypoxia-inducible factor 1α (HIF-1α) transcriptional activity, as well as an inhibition of histone deacetylase (*HDAC*) *6* and *9* gene expression [72]. Therefore, a better understanding of this mechanism could certainly contribute to the routine use of probiotic in FC treatment. A second potential mechanism of action might involve probiotic-mediated modulation of the gut microbiota–brain axis. In fact, studies carried out with animal models show that *L. reuteri* administration not only enhances the excitability of myenteric neurons, but also could interact with the enteric nervous system through afferent sensory nerves, thus improving gut motility [73,74]. Unfortunately, this mechanism has not yet been evaluated in patients suffering with FC. Lastly, the anti-inflammatory and antioxidant actions of probiotics might also represent new therapeutic options to treat FC. Through this mechanism, probiotics could potentially improve gut motility via the modulation of mucosal and systemic immune barrier integrity [75,76,77]. Again, further studies in humans are still required to confirm these potential properties in FC. As a consequence of this lack of scientific evidence, the routine use of probiotics in FC treatment is not currently recommended [52,58,78]. Likewise, it is important to note that a minority of gastroenterology specialists and general practitioners commonly recommend *L. casei* Shirota and VSL#3 (a combination of *Streptococcus*, *Bifidobacterium*, and *Lactobacillus*) as a coadjuvant treatment for FC; meanwhile, other probiotic strains such as *B. lactis* DN-173010, *L. casei* DN 111 001 and *L. casei* Shirota are regularly used by the general population [79]. Despite these promising results, there is currently no consensus on recommending the use of pro-, pre- or symbiotics as therapeutic strategies for the treatment of FC. In addition, the use of probiotics seems to be largely based on their availability and advertising rather than on evidence-based results. Both situations emphasize the need for good-quality RCTs that evaluate the potential beneficial effects of probiotics in infants and children with FC.

### 4.3. Potential Use of Probiotic Therapy in Other Pediatric FGIDs

As noted above, to date, probiotic therapy is only recommended in infant colic, while its clinical efficacy in pediatric FC is uncertain [52]. Similarly, scientific evidence to support probiotic treatment in other pediatric FGIDs, including irritable bowel syndrome (IBS), functional regurgitation and functional dyspepsia, is also limited.

Irritable bowel syndrome (IBS) is defined as a chronic disturbance of the gastrointestinal function characterized by upper and lower gastrointestinal symptoms as well as extra-intestinal symptoms, abdominal pain and altered intestinal routines [80]. IBS etiology remains unclear, but data obtained suggest potential involvement of gut microbiota dysbiosis. In fact, patients with IBS present a decrease in the *Bifidobacterium*/*Lactobacillus* ratio as well as an increase in the *Firmicutes*/*Bacteroidetes* ratio compared to healthy patients [81,82]. Consequently, the use of probiotics might enhance the gastrointestinal and health status of these patients. Thus, various meta-analyses and systematic reviews conclude that the use of different combinations of probiotics seems to be effective against IBS symptoms and related abdominal pain worldwide [81]. However, these conclusions should be taken with caution due to the existence of major limitations concerning the inclusion of studies with a small sample size and the use of different strains of probiotics [83]. Moreover, most of the referenced studies are performed in adult patients, and further efforts are still needed to support the clinical use of probiotics on pediatric IBS patients.

Functional regurgitation is a common FGID in the pediatric population, affecting up to 67% of infants during early life (before 4 months of age). This disorder is characterized by irritability, crying, food refusal and back arching, which negatively impact the infant’s quality of life [84]. In addition to these short-term effects, frequent regurgitation during infancy may also have long-term consequences for health, increasing the risk of heartburn, vomiting and acid regurgitation at 9 years of age [85]. Despite the high incidence in the pediatric population, there is a need for an evidence-based consensus to outline clinical recommendations for the treatment of functional regurgitation. Thus, on the one hand, pharmacotherapy is not recommended due to the lack of scientific evidence and potential risk of adverse events [86]. Similarly, conservative treatments, such as maintaining an upright position during the postprandial period, have been discouraged by ESPGHAN, NASPGHAN and the American Academy of Pediatrics guidelines because of the risk of sudden infant death syndrome [86,87,88]. To date, nutritional interventions based on thickened feedings and anti-regurgitation formulas, especially those with digestible carbohydrates, seems to be the primary treatment option for infants with functional regurgitation [89]. Interestingly, based on scientific evidence about therapeutic use of probiotics, experts suggest that this type of infant formula should be supplemented with *Lactobacillus reuteri* (now *Limosilactobacillus reuteri*) DSM 17938, enhancing its nutritional quality and gastric emptying rate in order to offer potential benefits in functional regurgitation treatment [90].

Finally, there is growing interest in specific clinical approaches that emphasize the prevention and treatment of pediatric functional dyspepsia (FD). This disorder can be defined as a complex of symptoms originating from the gastroduodenal region in the GIT, and is clinically characterized by epigastric pain and burning, postprandial fullness or early satiety. According to the presence or absence of these symptoms, FD is classified into postprandial distress syndrome (PDS), epigastric pain syndrome (EPS) and a subtype with overlapping PDS and EPS features [91]. Overall, the prevalence of functional dyspepsia is estimated to be around 20% of the general population, but to date the pathophysiological mechanisms involved are largely unknown [92]. Consequently, current treatments have limited efficacy or present major safety issues, and new therapeutic strategies are needed to improve treatment of dyspepsia. In this regard, although there is still little evidence of the efficacy of probiotics, prebiotics or synbiotics for FD, their potential use might improve the FD-related clinical outcomes. Thus, scientific evidence supports low-grade inflammation and increased duodenal mucosal permeability as key pathophysiological mechanisms involved in FD [93]. Therefore, the use of probiotics could improve mucosal permeability through the establishment of healthy gut microbiota and/or production of SCFAs. In fact, Wauters et al. [94] evaluated the potential role of spore-forming probiotics as a monotherapy or long-term add-on therapy with proton-pump inhibitors in patients suffering from FD. These authors reported that therapeutic strategies using *Bacillus coagulans* MY01 and *Bacillus subtilis* MY02 were a safe and effective way to treat FD. Conversely, a recent meta-analysis carried out by Zhang et al. [95] showed little evidence for the therapeutic use of pre- or synbiotics in FD, while the administration of probiotics alone failed to improve FD-related symptoms. Data obtained also suggested that the use of a combination of probiotics and prebiotics was more effective than other potential therapies, but further well-designed randomized clinical trials (RCTs) are still needed to provide the knowledge that should guide their therapeutic use in FD.

## 5. Potential Role of Probiotics in the Prevention and Treatment of Prevalent GIDs in Infants and Children

In this last subsection, the implication of gut microbiota dysbiosis in the most prevalent pediatric organic GIDs will be discussed, with a special focus on the available scientific evidence concerning the use of probiotics in their treatment and prevention.

### 5.1. Necrotizing Enterocolitis (NEC)

NEC is a deadly intestinal disorder that occurs predominantly in preterm very-low birth-weight infants during the first weeks of life, and it is characterized by intestinal inflammation and necrosis [96]. Due to its high morbidity and mortality in this population, there is growing scientific and clinical interest in not only better understanding the risk factors and pathophysiological mechanisms involved in NEC, but also diagnostic procedures and potential therapeutic interventions. In fact, NEC is a multifactorial disease caused by the interaction of multiple risk factors, including early gestational age, low birth weight, prolonged total parental nutrition or artificial milk feeding, among others, which ultimately can lead to gut microbiota dysbiosis and an immature immune response [97]. Consequently, early diagnosis of NEC should become a priority to reduce both its incidence and mortality. On the other hand, although its pathophysiology is currently poorly defined, scientific evidence seems to suggest that NEC is related to premature intestinal mucosa overactivation to bacterial antigens and subsequent mucosal destruction and rapid deterioration of mesenteric perfusion [98].

Taking into account all aspects previously mentioned, therapeutic strategies in NEC are largely based on gut dysbiosis prevention via implementation of standardized feeding protocols that favor breastmilk, moderate antibiotics use and probiotics administration [99]. Focusing on the therapeutic role of probiotics in NEC, a recent systematic review and meta-analysis of RCTs shows that *Lactobacillus acidophilus* LB had the most promising effect in reducing NEC risk in both breastfed and formula-fed preterm infants, while *Bifidobacterium lactis* Bb-12/B94 administration was associated with a reduced risk of mild/severe NEC [100]. Similar findings were obtained in other meta-analysis based on moderate-to-low-quality evidence [101]. More specifically, this work indicated that implementation of routine probiotic supplementation was associated with a reduced risk of mild/severe NEC, late onset sepsis and all-cause mortality in preterm infants. Moreover, this therapeutic strategy was also effective for mild/severe NEC in extremely low-birth-weight (<1000 g) neonates. Interestingly, other probiotic strains, such as *L. rhamnosus* GG ATCC53103 or the combination of *B. infantis* Bb-02, *B. lactis* Bb-12 and *Streptococcus thermophilus* TH-4, also seem to reduce NEC rates in preterm infants, but there is weak scientific evidence to support their general recommendation [102]. Overall, these findings should be taken with caution due to methodological limitations in the analysed studies, including relatively small populations; missing data about feeding practices and high variability in the probiotic strains used, doses and duration of the treatment [100,101]. Moreover, probiotic therapy must be used with particular care in preterm infants due to their immature gastrointestinal tract and undeveloped immune system, avoiding those probiotic strains with transferable antibiotic resistance genes or with the potential to cause sepsis [102]. Consequently, probiotic strains including *L. reuteri* DSM 17938, *B. breve* BBG-001, *Saccharomyces boulardii*, as well as the combination of *B. bifidum* NCDO 1453 and *L. acidophilus* NCDO 1748, are not recommended to treat NEC in this population.

While probiotics have shown encouraging results in NEC treatment, their precise mechanisms of action have not yet been elucidated. In this sense, data obtained from meta-analysis seem to support that probiotics modulate the systemic NF-κB-dependent inflammatory response associated with NEC, either acting on TLR4 receptor expression or reducing plasma endotoxin levels [103]. Interestingly, other studies suggest novel targets for probiotic treatment in NEC-associated inflammatory responses. For instance, Claud et al. [104] showed specific downregulation of inhibitor κB (*IκB*) expression in premature enterocytes, which might partly explain excessive NF-κB-mediated IL-8 production and subsequent pro-inflammatory status in NEC patients. On the other hand, in vitro analysis indicated that microbial DNA of *L. rhamnosus* HN001 could attenuate the NEC-associated inflammatory response through the TLR9 mediated signalling pathway, thus modulating dendritic and B cell-dependent acquired immunity [105]. In addition to this, the beneficial effects of probiotics on NEC also seem to be related to different action pathways [103]. Among these, probiotic treatment might enhance gut barrier function and integrity seriously harmed in NEC by increasing the synthesis of mucus, intercellular junction proteins and brush border enzyme. Interestingly, it is also proposed that probiotics decrease NEC-associated infections due to their ability to inhibit growth and viability of potential pathogens through mechanisms that involve competitive inhibition and synthesis of antimicrobial peptides and SCFAs. In relation to the aforementioned anti-inflammatory effects, probiotic administration could modulate oxidative stress and related apoptotic pathways, which are overexpressed in NEC patients. Finally, probiotics might regulate the activity of secretory epithelial Paneth cells, which secrete antimicrobial peptides and proteins that ultimately influence gut microbiota composition [103]. Recently, Zhao et al. [106] showed that probiotic effects on intestinal barrier function can also be related to the modulation of the Pregnane X Receptor (PXR)-c-Jun N-terminal protein Kinase (JNK) signalling pathway. In fact, in addition to its anti-inflammatory effects, these authors found that administration of a probiotic mixture based on *Bifidobacterium infants*, *Lactobacillus acidophilus*, *Enterococcus* and *Bacillus cereus* in an NEC mouse model increased PXR and tight junction component expression through the inhibition of JNK phosphorylation.

### 5.2. Helicobacter pylori Infection

*H. pylori* (HP) is a microaerophilic Gram-negative bacterium that causes infection of the mucosal layer of the stomach and/or duodenum [107]. This infection affects more than half of the world’s population, with a higher prevalence in developing countries [108]. Interestingly, HP infection is mainly acquired with no symptoms during early childhood, but usually causes severe gastroduodenal diseases such as peptic ulcer disease (PUD) and gastric cancer (GC) later in life [109]. Nonetheless, current data suggest that its incidence has considerably decreased in the child population due to better quality healthcare, improved standards of living and the low birth rate [110].

Despite its high prevalence and long-term consequences, many aspects relating to the pathophysiological mechanisms of HP infection are not fully understood. However, there is growing evidence supporting a potential link between HP infection and NF-κB-dependent chronic inflammation of gastric mucosa [111]. In fact, during its invasion and colonization of gastric mucosa, different HP antigens (lipoproteins, Heat Shock Protein (HSP) 60, lipopolysaccharide (LPS), lipoteichoic acid, NapA, RNA and DNA) are recognized by TLR receptors located on epithelial cell membranes and in intracellular vesicles [112]. Consequently, ligand–receptor binding triggers activation of the NF-κB and JNK signalling pathway, leading to an increase in the synthesis of proinflammatory cytokines and chemokines [113]. Simultaneously, the pro-inflammatory cytokine secretion is also enhanced by recognition of virulence factor CagA and subsequent activation of the bacterial-type secretion system (T4SS) and NF-κB pathway [113,114]. As a result, this activation cascade leads to cytokine overproduction and CD4+ helper T (Th)-17 cell hyperactivation that, in turn, results in more severe gastritis and higher prevalence of PUD and GC [111].

According to the ESPGHAN/NASPGHAN guidelines, HP infection diagnosis must be largely based on non-invasive techniques, such as serologic, urea breath or stool antigen tests [115]. These guidelines also support triple therapy with amoxicillin, clarithromycin and a proton pump inhibitor for 14 days to treat HP infection, as well as its monitorization after 4–8 weeks using reliable non-invasive tests. However, if its antimicrobial susceptibility is unknown or resistant to clarithromycin and metronidazole, bismuth-containing quadruple therapy is considered as the best therapeutic option. Despite these recommendations, and as a consequence of antibiotic resistance in HP levels increasing worldwide, novel therapeutic approaches, such as the potential use of probiotics, are still urgently needed in clinical practice. Thus, Losurdo et al. [116] carried out a systematic review to evaluate both the eradication rate and urea breath test delta value before and after probiotic monotherapy. These authors found that probiotic therapy eradicated HP infection in 14% of the selected population, although there were slight differences according to the probiotic strains used. In fact, *Lactobacilli*-based therapy was effective in 30 out of 235 patients (mean weighted rate of 16%), while the pooled eradication rate was 12% and 14% after probiotic treatment based on *Saccharomyces boulardii* and multi-strain formulations, respectively. Similar results were found in a recent meta-analysis, indicating that probiotic therapy in combination with classical treatments improved the eradication rate while reducing the total side effects associated with HP infection [117]. Several hypotheses have been proposed to explain the potential mechanisms of probiotic action in the treatment of HP infection. In vitro analysis showed that *L. acidophilus* and *L. bulgaricus* had the ability to inhibit HP adherence to human gastric epithelium GES-1 cells via the TLR4/IκBα/NF-κB pathway, thus modulating the IL-8-dependent inflammatory response [118]. In this line, Chen et al. [119] also observed that *Lactobacillus* spp. downregulated the pro-inflammatory NF-кB signalling pathway and subsequent production of inflammatory mediators such as IL-8, cyclooxygenase 2 (COX-2) and nitric oxide (NO). Interestingly, Lee et al. [120] found that *Lacticaseibacillus rhamnosus* and *Lactobacillus acidophilus* could trigger the suppression of cytokine signalling (SOCS)-dependent anti-inflammatory mechanisms, including phosphorylation of signal transducers and activation of transcription (STAT)-1 and STAT-3 protein family members as well as the inhibition of Janus kinase (JAK)2 phosphorylation. Overall, although further RCTs are still needed to better understand their mechanisms of action, routine use of probiotics as an adjuvant therapy is strongly recommended to enhance HP eradication and immune responses against HP-associated infection.

### 5.3. Role of Probiotics in Acute Infectious Diarrhea and Antibiotic-Associated Diarrhea in the Child Population

One of the most common diseases worldwide for those of a pediatric age is acute infectious diarrhea (AID), which is defined as “a specific microorganisms-mediated gastrointestinal infection that causes the passage of three or more loose or liquid stools per day, for 3 or more days, and less than 14 days, with or without additional symptoms such as nausea, vomiting, fever or abdominal pain” [121,122]. These symptoms usually last only a few days, without short- or long-term consequences. However, more severe and prolonged cases of diarrhea result in profound dehydration, weight loss and metabolic abnormalities that might require immediate hospitalization and could even be life-threatening [121,122]. From an aetiologic point of view, rotavirus has been identified as a primary cause of AID among children under 5 years of age, accounting for up to 40% of cases [123,124]. Additionally, pathogenic bacteria, mainly diarrheagenic *Escherichia coli*, *Salmonella* spp., *Shigella* spp., *Yersinia* spp., *Campylobacter jejuni*, *Clostridioides difficile* or *Vibrio cholera*, or parasitic infection by *Giardia* or *Cryptosporidium*, can also be involved in AID development [123]. Regardless of the causative agent of infection, epidemiologic studies also support that local (availability of clean water and sanitation) and host factors (abnormal intestinal motility and mucus secretion, gut microbiota dysbiosis or impaired immune response, among others) markedly influence infection-specific susceptibility [125].

Several hypotheses have been formulated regarding AID’s physiopathological mechanism. First, it is well-established that enteric pathogens involved in AID impaired secretion or absorption of fluids and electrolytes through lateral spaces between the intestinal epithelium cells or via certain ionic transporters [126]. Interestingly, the pathophysiology of AID is also closely related to specific virulence factors present in infectious agents, which cause different clinical manifestations associated with alternative mechanisms of action. Overall, enteric pathogens, including rotavirus, *V. cholerae* and enterotoxigenic *Escherichia coli*, have the ability to secrete enterotoxins in the small intestine. Once recognized by specific receptors on the surface of enterocytes, these enterotoxins trigger protein kinase activation, which ultimately leads to impaired electrolytes transport and subsequent non-inflammatory diarrhea episodes characterized by large amounts of watery stools and vomiting [125]. On the other hand, both non-invasive (enteroaggregative and enterohemorrhagic *Escherichia coli*, and *C. difficile*) and invasive enterogenic bacteria (*Shigella* spp., *Campylobacter* spp., *Salmonella* spp., *Yersinia* spp., enteroinvasive *Escherichia coli* and *Entamoeba histolytica*) cause inflammatory diarrhea, which is characterized as more severe and involves bloody, mucoid small-volume stools, abdominal pain and fever [125]. Despite their major differences, the abovementioned pathogens share the ability to colonize the lower bowel, an initial step toward the onset of diarrhea. Thus, after intestinal colonization, pathogenic bacteria secrete a wide variety of noxious cytotoxins or virulence intraluminal factors, which in turn results in the impairment of epithelial cells, altered fluid and ionic secretion, cell damage and subsequent host immune responses [125,127,128]. Finally, the enteric nervous system (ENS) seems to be also involved in AID pathophysiology via the secretion of different transmitters from the enterochromaffin cells into the lamina propria. Through afferent neurons, this event ultimately induces endogenous peptide secretion from goblet cells, which ultimately induces an imbalance of absorption and secretion of Cl^-^ and consequent diarrhea episodes [129,130].

Despite there being no specific treatment for AID, the current guidelines and recommendations strongly support oral rehydration based on reduced (Na^+^ 75 mmol/L) or hypotonic (Na^+^ 60 mmol/L) osmolarity solutions as first-line therapy for its clinical management [131]. These fluid management plans must be defined according to the patient´s hydration status. Additionally, specific dietary modifications should be implemented in those children who suffer from diarrhea with dehydration; in those cases, clinical practice guidelines recommend starting feedings 4–6 h after the onset of fluid management [122,131,132]. Moreover, breastfeeding should not be interrupted, and, in those formula-fed infants, it is not recommended to use diluted formula milk or gradually reintroduce it [122,131,132]. Novel complementary therapies using an oral rehydration solution should be largely based on the use of probiotics. In this sense, in 2023, the ESPGHAN Working Group on Probiotics and Prebiotics guidelines showed conditional recommendations for the therapeutic use of the following probiotic strains: (i) *Lacticaseibacillus rhamnosus* GG (a single dose of ≥10^10^ cfu/day for 5–7 days); (ii) *Saccharomyces boulardii* at a dose of 250–750 mg/day during 5–7 days; (iii) *Limosilactibacillus reuteri* DSM 17938 at daily doses ranging from from 1 × 10^8^ to 4 × 10^8^ cfu over 5 days; and (iv) combined treatment based on 2 × 10^10^ cfu of *L. rhamnosus* 19070-2 and *L. reuteri* DSM 12246 for 5 days [52]. Moreover, this paper strongly discourages the use of *Lactobacillus helveticus* R0052 and *L rhamnosus* R0011 as therapy options, as well as the treatment based on *Bacillus clausii* strains O/C, SIN, N/R and T, although to a lesser extent. Interestingly, *L. rhamnosus* GG is also recommended for the prevention of nosocomial diarrhea in the pediatric population [133]. These recommendations have also been supported by recent scientific literature. In fact, data obtained from RCTs support that LGG treatment reduced the duration of diarrhea episodes, stool number per day as well as the length of hospital stays, particularly in those patients infected with rotavirus. Interestingly, therapeutic outcomes were more effective with doses above 10^10^ cfuU/day and during the early stages of infection [134]. *Saccharomyces boulardii*-based therapy is also recommended as a promising primary or adjuvant strategy against infant diarrhea due to its potential beneficial effects on the duration of diarrhea episodes, hospitalizations and vomiting [135]. Finally, inconclusive results have been obtained for the use of *L. reuteri* DSM 17938 for treating AID in children [136,137]. However, results present here should be viewed with caution due to the lack of high-quality RCTs that evaluate the role of probiotics on diarrhea, as well as important methodological limitations, including an unknown aetiology, small sample size, different therapeutic strategies or the absence of a standard clinical outcome [123,135,136,137,138]. Consequently, other Working Groups, such as the American Gastroenterological Association and Cochrane Infectious Diseases Group, did not support the use of probiotics as a potential treatment of infant AID [123,138]. This inconclusive evidence highlights the need for further studies to better understand the potential of the therapeutic use of probiotics. Despite latter inconsistencies or contradictions, clinical perspectives for the use of probiotics in AID treatment have gained great interest due to their potential direct and indirect antimicrobial mechanisms of action [139]. Among them, probiotics could exert direct and early beneficial effects on intestinal mucosa integrity to reduce its colonization by enteropathogens. These direct effects are associated with competition for both nutrients and binding to cell surface receptors, neutralization of pathogen-derived toxins, as well as antimicrobial compound secretion such as bacteriocins and metabolites, particularly SCFAs [139]. Interestingly, these compounds also seem to show postbiotic effects through which they might modulate oxidative stress and anti-inflammatory pathways, thus preventing pathogens-associated enterotoxic and cytotoxic damage [140]. Additionally, probiotics also exhibit indirect, late but lasting effects against enteropathogens, including: (i) an improvement in intestinal barrier function via the upregulation of mucins and tight junctions proteins expression; (ii) the high production of beta-defensin, a peptide with broad antibacterial activity; (iii) gut microbiota modulation; and finally, (iv) immune response modulation, mainly due to the increasing IgA production related to the maturation and/or activation of dendritic cells, B lymphocytes and T cells, and in response to the production of transforming growth factor β (TGF-β), IL-6 and IL-10 [139].

Finally, it is important to note that antibiotic therapy for AID is not recommended to be given routinely, except for specific pathogens (*Shigella*, enterotoxigenic *E. coli* and *Vibrio cholerae*), or in defined clinical conditions [131]. In fact, widespread and inappropriate use of antibiotics can alter gut microbiota composition and function as well as epithelial barrier integrity, thus promoting overgrowth of opportunistic enteropathogens and subsequent intestinal colonization [141]. Consequently, a common and challenging complication is the appearance of antibiotic-associated diarrhea (ADD). This disorder is particularly prevalent in patients with chronic conditions that ultimately lead to fulminant pseudomembranous colitis caused by *Clostridioides difficile* and, to a lesser extent, by *Staphylococcus aureus* and *Clostridium perfringens* [142]. Due to its aetiology, the withdrawal of antibiotic therapy is obviously the first step that should be taken towards its treatment, followed by oral rehydration solutions and a healthy diet. These therapeutic actions are focused on preventing a catabolic state as well as promoting enterocyte regeneration. If the response is not satisfactory, or in most severe cases of ADD, it is recommended that treatment be based on oral metronidazole or vancomycin and given for at least one week [141]. Despite the efficacy of these therapeutic practices, about 5–30% of patients may experience recurrence of ADD within 1–2 weeks, indicating a clear need to evaluate other potential treatments. According to their direct and indirect mechanisms of action on intestinal barrier function and diarrhea-related disorders, probiotics have emerged as promising tools for the prevention and treatment of ADD. In fact, based on the moderate quality of the evidence, the ESPGHAN Working Group on Probiotics strongly recommended the use of *Lacticaseibacillus rhamnosus* GG and *Saccharomyces boulardii* for ADD prevention in children. Interestingly, *S. boulardii*-based treatment appears to be the most effective for preventing *Clostridioides difficile*-related diarrhea [142]. It is also important to note that there is a lack of evidence to support the therapeutic use of other probiotic strains such as *Bacillus clausii* and different mixes of probiotics, including *Bacillus lactis*/*Streptococcus thermophilus*, *L. acidophilus*/*L. bulgaricus* and *L. acidophilus*/*Bifidobacterium infantis*, among others [142]. Data obtained from recent systematic reviews also support the abovementioned recommendations [143,144]. However, despite these positive and encouraging results, large, well-designed multi-center RTCs are still needed to determine the optimal daily doses of probiotics, their safety for preventing ADD in vulnerable populations, as well as the safety and beneficial clinical effects of other probiotic strains.

### 5.4. Inflammatory Bowel Disease

Inflammatory bowel disease (IBD) is an immune-mediated disease that is classified into ulcerative colitis (UC), Crohn’s disease (CD) and unclassified IBD (U-IBD) [145]. It is caused by an exaggerated mucosal immune response to gut microbiota in genetically predisposed hosts mediated by several genetic, immune and environmental factors [146,147]. Additionally, an alternative hypothesis also suggests that a diet high in sugar, fat and fiber could contribute to the development of the altered immune response found in IBD patients [148].

Regardless of the abovementioned classification, IBD has an increasing incidence in the pediatric population. The most common symptoms in children are weight loss, abdominal pain, bloody diarrhea, poor growth, anemia and other extra-intestinal manifestations [149]. Once IBD is diagnosed, the main therapeutic goals must be to eliminate symptoms, thereby normalizing quality of life, restoring healthy growth and development, and preventing complications, while minimizing the adverse effects of medications [150]. Interestingly, in more recent years, due to clinical advances in IBD pathophysiology, therapeutic strategies acquire a personalized profile based on the knowledge obtained from different clinical outcomes, including the severity of IBD, its location, patient phenotype, potential effects of IBD on growth and development, as well as the psychological state of the patient [151]. This therapeutic strategy usually consists of a stepped treatment in which aminosalicylates, antibiotics, enteral therapy and biological immuno-modulators are used. Moreover, ”step-up” treatment is also effective in acute cases in which surgery is essential [152]. Together these therapeutic actions, and according to the ESPGHAN positional paper, nutritional interventions should also be implemented by physicians and nutritionist to improve care for pediatric IBD patients [153]. Among them, it is crucial to clinically monitor vitamin D levels, use iron supplements to prevent and treat iron deficiency anemia, and oversee treatment with folic acid (either 1 mg/day or 5 mg/day) and subsequent annual monitoring of its levels in those children with IBD receiving methotrexate therapy, as well following the use of a standard polymeric formula with a moderate fat content.

From a clinical point of view, IBD-related microbial dysbiosis, in combination with modified dietary habits, leads to altered levels of microbial metabolites, which in turn induce marked changes in host metabolism and functions. Despite the use of probiotics being effective for the treatment of colitis in animal models [154], their therapeutic effects in IBD clinical trials have been less satisfactory, with very specific beneficial effects in certain patients [155]. This may be in part due to the administration of probiotics without first identifying the existence of environmental and immunological niches capable of accommodating them, thus altering the ability of probiotic strains to establish the GIT of patients. Moreover, exaggerated inflammation related to IBD can also impair the potential beneficial effects of probiotic-based strategies [150]. To date, the significant efficacy of probiotic preparation of VSL#3 has only been reported in the child population (mean age 12), with mild-to-moderate UC [156]. In light of these findings, major efforts are still needed to design optimal probiotic strategies that overcome the aforementioned limitations. Among them, increasing the knowledge surrounding the composition of the human microbiome will allow us to identify novel potential probiotic species that adequately colonize the human intestinal tract. Their beneficial effects will largely depend on the initial microbiota of the individual, as well as the availability of nutritional resources; moreover, the role of dietary substrates such as fermentable carbohydrates and prebiotics will also be essential in allowing for the long-term persistence of a probiotic. In fact, prebiotics could create novel nutritional niches within the human gastrointestinal tract, also regulating intestinal immunity by releasing metabolites with beneficial effects on the intestinal microbiota [150,157]. However, to date, few prebiotic interventions have been performed in patients with IBD, and results obtained suggest neither adverse effects nor clinical improvement in these patients. Similar to the use of probiotics, individualized prebiotic interventions in terms of type, dose and administration guidelines must be essential in order to obtain promising results in these patients [158].

### 5.5. Role of Probiotics in Food Allergies

In recent years, there have been significant advances in understanding the role of intestinal microbiota in food allergy (FA) development, as well as how targeted bacterial therapies may be effective in its prevention and treatment [159]. For instance, differences in gut microbiome composition of a child between the age of 3–6 months seems to be related to the acquisition of tolerance to milk proteins by the age of 8 [160]. At this age, the gut microbiota from infants with a resolved cow milk allergy are characterized by a high abundance of *Clostridia* and *Firmicutes*, thus emerging as potential probiotic strains to be used in milk allergy therapy [160].

Diverse novel therapeutic strategies focused on allergen- and non-allergen-specific therapies have been developed, but further efforts are still needed to implement them at the clinical level [161]. Among these latter strategies, probiotic-based therapy has gained considerable interest over the last decade, particularly due to its ability to modulate intestinal barrier development and maturation [162]. Additionally, therapeutic use of probiotics in FA is also supported by their immunomodulatory effects, including Th1 production and Th2 suppression, tolerogenic dendritic cell development, the suppression of IgE production as well as the epigenetic modulation of *Th1/Th2* gene expression [162]. Interestingly, recent interest has emerged concerning the potential use of new probiotic-related concepts such as postbiotics in FA treatment, which may be defined as “bioactive soluble factors (products or metabolic by products) obtained from probiotic cultures showing biological activity in the host without risks associated with the use of live probiotic strains” [163]. In fact, both in vitro and in vivo studies have shown that therapeutic approaches based on postbiotics (mainly SCFAs such as acetate, butyrate and propionate) might be effective in FA due to the enhancement of epithelial barrier integrity. This beneficial effect is mediated through diverse mechanisms of action, including: (i) an upregulation of genes related to tight-junctions components; (ii) the activation of the STAT3 and SP1 pathways related to protein reassembly; (iii) the generation of antimicrobial peptides via IECs; and (iv) an increase in transepithelial electrical resistance [164,165,166,167]. Additionally, both pro- and postbiotics may also maintain gut epithelial barrier integrity via the activation of group 3 innate lymphoid cells (ILC3) and subsequent IL-22 production, thus promoting mucus and antimicrobial peptide secretion by goblet and Paneth cells, respectively. Consequently, these changes might lead to the decreased accessibility of dietary antigens to the host systemic circulation and, therefore, reduce food allergen sensitization [168].

Despite advances in understanding their potential benefits in FA treatment, the routine use of probiotics for preventing this disorder is not currently recommended, and further well-powered studies are still needed. In fact, updated guidelines from the European Academy of Allergy and Clinical Immunology (EAACI) did not provide recommendations in favor of or against therapeutic use of probiotics in FA [169]. This guideline primarily exposes that the clinical effects and level of safety observed in the use of any single probiotic, or a combination of probiotics, prebiotics or synbiotics, cannot be extrapolated to other probiotics strains. These extrapolation problems are not only due to immunological differences between strains used, but also because studies performed largely differ in terms of the size population, duration, type and timing of the probiotic therapy, diagnostic criteria and follow-up duration.

## 6. Conclusions and Perspectives

Both functional and organic GIDs are very common in the pediatric population, and their short- and long-term consequences reveal many challenges for physicians and pediatricians. Recently, scientific and clinical interest has focused on evaluating the potential adverse effects of GIDs on the composition and function of the gut microbiota as a cause or consequence of these comorbidities. In this review, we synthesized the available scientific evidence about relationships between gut microbiota dysbiosis and clinical outcomes in pediatric patients suffering from GIDs. Based on this, and according to their different and complex mechanisms of action, a form of therapy based on the use of probiotics has emerged as a promising strategy for the prevention and treatment of several GIDs. There is growing evidence that probiotic treatment promotes a protective environment for commensal bacteria and generates an interface for immune response, thus improving clinical outcomes in pediatric patients with GIDs. Furthermore, the safety of the use of probiotics has been well-established in clinical trials, with adverse effects only reported in patients with compromised health. Unfortunately, a broad consensus for most of indications, specific strains, dosages and treatment regimens is lacking.

To overcome these limitations, well-designed and well-powered studies are still needed, which should be focused on: (a) identifying specific gut microbiota dysbiosis patterns related to GIDs; (b) providing better understanding of the role of probiotics and other gut microbiota-based treatments (pre-, synbiotics, postbiotics and/or paraprobiotics) as therapeutic agents and their mechanisms of action on gut microbiota; and (c) extrapolating findings obtained in animal models to humans population, particularly in the pediatric population. This new knowledge must not only be considered in unison with updated guidelines to recommend the use of probiotics as an adjunctive therapy in the prevention and treatment of pediatric GIDs, but also to design individualized strategies in this population, improving their clinical outcomes and subsequently reducing the global burden of these diseases.

## Figures and Tables

**Table 1:** Oxford Centre for Evidence-Based Medicine levels of evidence for treatment benefits relative to the question “Does this intervention help?”.

Evidence Level	Study Type
1 *	Systematic review of randomized trials
2 *	Randomized trial with consistent effect, without systematic review
3 *	Supported by a single randomized controlled trial ^†^
4	Case-series, case–control studies, or historically controlled studies ^†^
5	Mechanism-based reasoning

Reprinted by permission from the World Gastroenterology Organisation [8]. * The level may be downgraded on the basis of study quality, imprecision, and indirectness—the study’s population, intervention, comparison and outcome (PICO) criteria do not match the question’s PICO; because of inconsistency between studies; or because the absolute effect size is very small. The level may be upgraded if there is a large or very large effect size. ^†^ A systematic review is considered to provide higher-quality evidence than an individual study.

**Table 2:** Evidence-based pediatric indications for the use of probiotics, prebiotics and/or synbiotics in gastroenterology.

Disorder, Action	Probiotic Strain/Prebiotic/Synbiotic	Recommended Dose	Evidence Level *	Comments
Acute Gastroenteritis	Probiotics as a general group	N/A	1	Reduced the risk of diarrhea lasting ≥ 48 h; reduced the mean duration of diarrhea (based on an updated Cochrane review including 82 RCTs (*n* = 12,127 participants), mainly in children (*n* = 11,526)
	*L. rhamnosus* (now *Lacticaseibacillus rhamnosus*) GG	≥10^10^ cfu/day, for 5–7 days	1	Reduced duration of diarrhea, length of hospitalization and stool output. ESPGHAN 2022
	S. boulardii **	250–750 mg/day, for 5–7 days	1	Reduced duration of diarrhea. ESPGHAN 2022
	*Lactobacillus reuteri* (now *Limosilactobacillus reuteri*) DSM 17938	1 × 10^8^ to 4 × 10^8^ cfu/day, for 5 days	1	Reduced duration of diarrhea. ESPGHAN 2022
	*L. rhamnosus* 19070-2 & *L. reuteri* DSM 12246	2 × 10^10^ cfu for each strain/day, for 5 days	1	Reduced duration of diarrhea. ESPGAHN 2022
	*B. lactis* B94 + inulin	5 × 10^10^ cfu plus 900 mg once daily, respectively, for 5 days	3	Reduced duration of acute watery diarrhea
	*Lactobacillus paracasei* (now *Lacticaseibacillus paracasei*) B21060, plus arabinogalactan, and xylooligosaccharides	2.5 × 10^9^ cfu plus 500 mg plus 700 mg, respectively, twice daily, for 5 days	3	Reduced duration of diarrhea
	*L. rhamnosus* strains 573L/1; 573L/2; 573L/3	1.2 × 10^10^ cfu or placebo, twice daily, for 5 days	3	Reduced duration of rotaviral diarrhea, but not of diarrhea of any etiology
	*L. delbrueckii* var. *bulgaricus*, *L. acidophilus*, *Streptococcus thermophilus*, *B. bifidum* (LMG-P17550, LMG-P 17549, LMG-P 17503, LMG-P17500)	10^9^ cfu, 10^9^ cfu, 10^9^ cfu, 5 × 10^8^ cfu/dose, for 5 days	3	Reduced duration of diarrhea
	*B. lactis* Bi-07, *L. rhamnosus HN001*, and *L. acidophilus* NCFM	Then, 1.0 × 10^10^ cfu once a day, for the duration of diarrhea plus 7 days	3	Reduced duration of diarrhea and reduced hospital stay
Prevention of Antibiotic-Associated Diarrhea	Probiotics as a general group	N/A	1	Reduced risk of AAD (a 2019 Cochrane review; 33 RCTs involving 6352 participants)
	*S. boulardii* **	≥5 billion cfu per day, for the duration of antibiotic treatment	1	Reduced risk of AAD/diarrhea. ESPGHAN 2016 and 2022
	*L. rhamnosus* GG	≥5 billion cfu per day, for the duration of antibiotic treatment	1	Reduced risk of AAD/diarrhea. ESPGHAN 2016 and 2022
	Multispecies probiotic (*Bifidobacterium bifidum* W23, *B. lactis* W51, *Lactobacillus acidophilus* W37, *Lactobacillus acidophilus* W55, *Lacticaseibacillus paracasei* W20, *Lactiplantibacillus plantarum* W62, *Lacticaseibacillus rhamnosus* W71 and *Ligilactobacillus salivarius* W24)	10 billion cfu per day, for the duration of antibiotic treatment and for 7 days after	3	Reduced risk of diarrhea but not AAD. The definition of diarrhea/AAD matters
	*L. rhamnosus* (strains E/N, Oxy, and Pen)	2 × 10^10^ cfu, twice daily, for the duration of antibiotic treatment	3	Reduced risk of diarrhea
Prevention of *C. difficile* Diarrhea	*S. boulardii* **	250–500 mg	1	ESPGHAN 2016 and 2022; AGA 2020; reduced risk of *C. difficile*-associated diarrhea
Prevention of Nosocomial Diarrhea	* L. rhamnosus * GG	At least 10^9^ cfu/day, for the duration of the hospital stay	1	ESPGHAN 2022; reduced risk of nosocomial diarrhea
Prevention of Necrotizing Enterocolitis	Systematic reviews and meta-analyses (>10,000 neonates) of RCTs		1	Some specific strains of probiotic may be effective for preventing NEC among preterm infants
	*L. rhamnosus GG*	1 × 10^9^–6 × 10^9^ cfu	1	ESPGHAN 2020 and 2022; AGA 2020
	*B. infantis* BB-02, *B. lactis* BB-12, and *S. thermophilus* TH-4	3.0 to 3.5 × 10^8^ cfu (of each strain)	1	ESPGHAN 2020 and 2022
	*B. animalis* subsp. *lactis* Bb-12 or B94	5 × 10^9^ cfu	3	
	*L. reuteri* ATCC 55730 or DSM 17938	1 × 10^8^ cfu (various regimens)	1	ATCC 55730; this strain is no longer available. Recommended by AGA 2020, but not ESPGHAN 2020 or 2022
	*B. longum* subsp. *infantis* ATCC 15697 *+ L. acidophilus* ATCC 4356	125 mg/kg/dose twice daily with breast milk until discharge	3	
	*B. longum* subsp. *longum* 35624 + *L. rhamnosus* GG	5 × 10^8^ cfu each strain	3	
*Helicobacter pylori* Infection	Probiotics as a general group		1	Improved eradication rates and/or reduced side effects of anti-*H. pylori* treatment
	*S. boulardii* **	500 mg	1	Increased eradication rate (however, it was still below the desired level [≥90%] success), and in reducing gastrointestinal adverse effects associated with *H. pylori* infection therapies. ESPGHAN 2022
	*Lactobacillus* (now *Lactiplantibacillus*) *plantarum* (UBLP 40), *L. acidophilus* (LA-5), *B. animalis* subsp. *lactis* BB-12 and *S. boulardii* Unique-28	Per capsule: *L. plantarum* (0.5 × 10^9^ cfu), *L. acidophilus* LA-5 (1.75 × 10^9^ cfu), BB-12 (1.75 × 10^9^ cfu) and *S. boulardii* (1.5 × 10^9^ cfu), twice daily for 15 days	3	Increased eradication rate and decreased side effects
	Fermented milk containing *L. casei* DN-114 001	10^10^ cfu/day for 14 days	3	
Infantile Colic	Probiotics as a general group	N/A	1	
Infantile Colic—Management	*L. reuteri* DSM 17938	10^8^ cfu/day for at least 21 days	1	Reduced crying and/or fussing time in breastfed infants, but its role in formula-fed infants is less clear. ESPGHAN 2022
	*B. lactis* Bb12	10^8^ cfu/day, for 21–28 days	2	Reduced crying and/or fussing time in breastfed infants with infantile colic. ESPGHAN 2022
	*L. rhamnosus * 19070-2 and *L. reuteri* 12246, fructooligosaccharide (FOS)	250 × 10^6^ cfu, respectively, plus 3.33 mg of FOS, daily dose, for 28 days	3	Reduced crying and/or fussing time in breastfed infants
	*L. paracasei* DSM 24733, *L. plantarum* DSM 24730, *L. acidophilus* DSM 24735, *L. delbrueckii* subsp. *bulgaricus* DSM 24734, *B. longum* DSM 24736, *B. breve* DSM 24732, *B. infantis* DSM 24737 and *S. thermophilus* DSM 24731	5 billion cfu, for 21 days	3	Reduced crying in exclusively breastfed infants
Infantile Colic–Prevention	*L. reuteri* DSM 17938	10^8^ cfu/day to newborns for 90 days	1	Reduced crying time in both breast-fed and formula-fed infants
Functional Abdominal Pain Disorders		N/A	1	No firm evidence for the use of probiotics (as a group) in children with FAPD
Functional Abdominal Pain/IBS	*L. reuteri* DSM 17938	10^8^ cfu to 2 × 10^8^ cfu/day	1	ESPGHAN 2022
	*L. rhamnosus* GG	10^9^ cfu to 3 × 10^9^ cfu twice daily	1	ESPGHAN 2022
Ulcerative Colitis	Probiotics as a group	N/A	1	May induce clinical remission in patients with active ulcerative colitis
	A mixture of 8 strains (*L. paracasei* DSM 24733, *L. plantarum* DSM 24730, *L. acidophilus* DSM 24735, *L. delbrueckii* subsp. *bulgaricus* DSM 24734, *B. longum* DSM 24736, *B. infantis* DSM 24737, *B. breve* DSM 24732 and *S. thermophilus* DSM 247), as adjuvant therapy or in those intolerants to 5-ASA	Daily dosages: 4–6 years (17–23 kg), 1 sachet (450 billion) 7–9 years (24–33 kg), 2 sachets (900 billion) 11–14 years (34–53 kg), 3 sachets (1350 billion) 15–17 years (54–66 kg), 4 sachets (1800 billion)	3	For induction and maintenance of remission. ESPGHAN & ECCO 2018
	*Escherichia coli* Nissle 1917 (as adjuvant therapy or in those intolerants to 5-ASA)	200 mg/day (in adults and adolescents; no dosing is available for young children)	3	For induction and maintenance of remission. ESPGHAN & ECCO 2018
Pouchitis	A mixture of 8 strains (*L. paracasei* DSM 24733, *L. plantarum* DSM 24730, *L. acidophilus* DSM 24735, *L. delbrueckii* subsp. *bulgaricus* DSM 24734, *B. longum* DSM 24736, *B. infantis* DSM 24737, *B. breve* DSM 24732 and *S. thermophilus* DSM 247)	Daily dosages: 4–6 years (17–23 kg), 1 sachet (450 billion) 7–9 years (24–33 kg), 2 sachets (900 billion) 11–14 years (34–53 kg), 3 sachets (1350 billion) 15–17 years (54–66 kg), 4 sachets (1800 billion)	3	Maintaining remission (but in adult patients) with chronic pouchitis. ESPGHAN & ECCO 2018 and AGA 2020
Non-Alcoholic Fatty Liver Disease	*Lactobacillus acidophilus* in combination with other strains of *Bifidobacterium* or *Lactobacillus* may be beneficial for improving levels of transaminases and lipid parameters as well as ultrasonographic and anthropometric characteristics in children with NAFLD. However, current evidence does not allow for specification of the exact beneficial strain of probiotic		1	

Reprinted by permission from the World Gastroenterology Organisation [8]. See original paper for more details. * Oxford Centre for Evidence-Based Medicine levels of evidence (see Table 1). ** Most studies with the strain *S. boulardii* CNCM I-745. AAD, antibiotic-associated diarrhea; AGA, American Gastroenterological Association; cfu, colony-forming unit(s); ECCO, European Crohn’s and Colitis Organization; ESPGHAN, European Society for Paediatric Gastroenterology, Hepatology, and Nutrition; FAPD, functional abdominal pain disorder; IBS, Intestinal Bowel Disease; LGG, *L. rhamnosus* GG; N/A, not available; NEC, necrotizing enterocolitis; RCT, randomized controlled trial.

## Data Availability

Not applicable.
